# Trends in fluoroquinolone resistance in *Campylobacter*

**DOI:** 10.1099/mgen.0.000198

**Published:** 2018-07-19

**Authors:** Emma L. Sproston, Helen M. L. Wimalarathna, Samuel K. Sheppard

**Affiliations:** ^1^​ The Milner Centre for Evolution, Department of Biology and Biochemistry, University of Bath, Bath BA27AY, UK; ^2^​ School of Medicine University of Central Lancashire, Preston, Lancashire PR1 2HE, UK

**Keywords:** *Campylobacter*, fluoroquinolones, ciprofloxacin, resistance

## Abstract

Members of the genus *Campylobacter* remain a leading cause of bacterial gastroenteritis worldwide. Infection is usually self-limiting but in severe cases may require antibiotic treatment. In a recent statement by the World Health Organization (WHO) *Campylobacter* was named as one of the 12 bacteria that pose the greatest threat to human health because they are resistant to antibiotics. In this mini review we describe recent trends in fluoroquinolone (FQ) (particularly ciprofloxacin) resistance in strains of members of the genus *Campylobacter* isolated from livestock and clinical samples from several countries. Using evidence from phenotyping surveys and putative resistance prediction from DNA sequence data, we discuss the acquisition and spread of FQ resistance and the role of horizontal gene transfer and describe trends in FQ-resistance in samples from livestock and clinical cases. This review emphasises that FQ resistance remains common among isolates of members of the genus *Campylobacter* from various sources.

## Data Summary

All external data records cited in the text of this review are listed in the bibliography at the end of the document.

**Impact Statement**

Fluoroquinolones (FQ), especially ciprofloxacin (CIP), are among the most common treatments for human and animal infection with *Campylobacter –* a common cause of gastroenteritis worldwide. Over time, this has resulted increased antimicrobial resistance (AMR), making these drugs much less effective. Here we review current literature to describe: (i) the mechanisms of FQ resistance in members of the genus *Campylobacter*; (ii) acquisition and spread; (iii) predicting resistance from whole-genome data; (iv) CIP resistance among clinical isolates; (v) CIP resistance in livestock; (vi) the potential for spread of resistance from animals to humans.

## Introduction

Members of the genus *Campylobacter* are the leading cause of bacterial gastroenteritis in many countries [1, 2]. They are frequently isolated from the gut of warm-blooded animals (particularly poultry) [1, 2] and the common pathogenic species *Campylobacter jejuni* (approximately 90 %) and *Campylobacter coli* (approximately 10 %) [3, 4] cause infection in humans, most commonly after consumption of contaminated or under-cooked food, especially poultry. Campylobacteriosis is usually self-limiting and rarely requires antibiotic treatment, except in severe or prolonged cases [2, 5]. The most common drugs used to treat *Campylobacter* infections are macrolides (particularly erythromycin) for laboratory-confirmed cases. Members of the genus *Campylobacter* are generally susceptible to erythromycin and in Europe gentamicin (an aminoglycoside) is the antibiotic to which they have the second lowest antimicrobial resistance (AMR) rates, after gentamicin [6]. The fluoroquinolones (FQ) are broad-spectrum antimicrobials that are used to treat a multitude of infections including undiagnosed cases of diarrhoea, predominantly by using ciprofloxacin (CIP) [2, 5, 7–10].

Since the late 1980’s there has been an increasing trend in the proportion of FQ-resistant strains of members of the genus *Campylobacter* isolated from both clinical samples [11–13] and livestock, where FQs (particularly enrofloxacin) are frequently used to treat animals in intensive production [5, 7, 9, 12, 14]. In general it appears that resistance to FQs, and several other antimicrobials (AM’s), is more common in *C. coli* compared with *C. jejuni* (Table 1) [4, 6, 7, 15, 16] but the reason for this is not fully understood. However, FQ resistance remains common in both species [17]. The widespread acquisition of FQ resistance is the result of spontaneous independent mutations and is accelerated by the horizontal transfer of resistance-conferring DNA among strains of members of the genus *Campylobacter* [5, 18]. In response to the rising levels of FQ and other AMR, restrictions on the use of FQs in animal husbandry have been implemented in the EU (2003 and 2006) and the USA (2005) [13, 19, 20], and several specific government-led AMR surveillance and monitoring programs have been initiated [21]. However, comprehensive data assessing CIP resistance in members of the genus *Campylobacter* has only become available in recent years [6, 15] and FQs are already of limited use for treating infections with members of the genus *Campylobacter* in many countries [5]. The primary FQ in *Campylobacter* AMR testing schemes is CIP, probably due to its common use in the treatment of diarrhoea. In Europe, CIP is the only FQ listed, and is a mandatory AM to be tested under the harmonised methods scheme for the monitoring of AMR in isolates of members of the genus *Campylobacter* from humans [22]. For isolates of members of the genus *Campylobacter* obtained from the major food animals, nalidixic acid (NAL) (a quinolone) is also included on the mandatory list of AM’s to be tested for resistance [23]. Many other studies follow this, where other FQ’s are rarely included in the AMR testing of *Campylobacter* isolates. However, the resistance to quinolone and fluoroquinoline have a strong correlation [9]. For example, a study has revealed that 69.8 % of isolates of *C. jejuni* from poultry were resistant to CIP and 65.1 % were resistant to NAL [6]. In other studies, isolates of members of the genus *Campylobacter* had similar proportions that were resistant to CIP and NAL [16, 24, 25]. This is most likely to be due to a high level of resistance being conferred by just a single point mutation within the quinolone-resistance-determining region (QRDR) and it being the most common and recognised mechanism for FQ resistance in members of the genus *Campylobacter* [5, 7, 14]. If an isolate has developed a high level of resistance to a FQ drug, it is likely to have similar resistance levels to other FQ’s.

**Table 1.** Ciprofloxacin (CIP) resistance in members of the genus *Campylobacter* from clinical, poultry and livestock samples

**Sample**	**Country**	**Area**	**Source**	**Sample type**	**Isolates year(s)**	Species*	**Resistance to CIP [% (*n*)]**		**Ref.**
**Clinical**	Canada	Montreal	Human		2002	*Cj*	41.6	(440)	[17]
					2013	*Cc*	50.0	(38)	
	Canada	Ontario	Human		2011	*Cj*	30.8	(180)	[53]
					2013	*Cc*	41.0	(39)	
	USA	Selected states	Human		2011	*Cj*	25.8	(5048)	[54]
					2015	*Cc*	36.3	(576)	
	USA	USA	Human		2011–12	*C.* Spp.	25.3	(1962)	[55]
	Peru	Lima	Human	Infant faeces	2008–11	*Cj*	87.0	(69)	[57]
						*Cc*	91.3	(46)	
	Europe	13 countries	Human		2014	*Cj*	60.2	(11 585)	[6]
						*Cc*	68.9	(1500)	
	Europe	17 countries	Human		2015	*Cj*	60.8	(13 696)	[15]
		16 countries				*Cc*	70.6	(1754)	
	France		Human		2014	*Cj*	55.6	(1997)	[56]
					2015	*Cc*	64.4	(419)	
	Poland	Bydgoszcz	Human	Child faeces	2011–13	*Cj*	65.2	(92)	[63]
						*Cc*	71.4	(7)	
	UK	Oxfordshire	Human		2008	*C.*spp.	37.5	(803)	[39, 58]
	China	Beijing	Human		1994/2010	*Cj*	86.7	(203)	[25]
**Broilers**	Africa	Kenya	Chicken	Faeces/cloaca		*Cj*	71.0	(31)	[24]
						*Cc*	75.0	(4)	
	China	Multiple	Chicken	Caecae	2008–09	*Cj*	99.4	(971)	[46]
		Multiple			2012–14	*Cc*	99.2	(1021)	
	China	Central	Chicken	Faeces/cloaca	2012–16	*Cj*	100.0	(166)	[61]
						*CC*	100.0	(40)	
	Europe	25 countries	Chicken	Caecae	2014	*Cj*	69.8	(3317)	[6]
		8 countries				*Cc*	74.3	(767)	
	Italy		Chicken	Cloaca/carcass	2014	*Cj*	39.0	(99)	[62]
					2015	*Cc*	69.7	(41)	
**Poultry meat**	China	Central	Chicken	Frozen/fresh	2014	*Cj*	100.0	(40)	[64]
					2015	*Cc*	100.0	(12)	
	Europe	3 countries	Chicken	Various	2014	*Cj*	65.6	(308)	[6]
						*Cc*	85.8	(134)	
		2 countries	Turkey	Slaughter/retail	2014	*Cj*	66.2	(74)	[6]
	Poland	Bydgoszcz	Poultry	Slaughter/retail	2011	*Cj*	62.2	(90)	[63]
					2013	*Cc*	74.1	(54)	
	UK	Multiple	Chicken	Retail	2014	*Cj*	49.1*	(230)	[16]
					2015	*Cc*	54.7*	(53)	
**Ruminants**	Africa	Ghana	Cattle	Faeces/carcasses	2013–14	*C.* spp.	42.6	(54)	[60]
			Sheep	Faeces/carcasses	2013–14	*C.* spp.	32.8	(64)	[60]
			Goat	Faeces/carcasses	2013–14	*C.* spp.	47.4	(57)	[60]
	USA	Michigan	Cattle	Faeces	2012	*Cj*	16.3	(22)	[40]
	USA	5 States	Cattle	Faeces	2012–13	*Cj*	35.4	(320)	[33]
						*Cc*	74.4	(115)	
**Swine**	China	Multiple	Pig	Faeces	2008–14	*Cc*	97.0	(970)	[39]
	Africa	Ghana	Pig	Faeces/carcasses	2013–14	*C.* spp.	30.3	(66)	[55]
	Europe	7 countries	Pig	Caecae	2015	*Cc*	62.1	(704)	[13]

*Cj (*C. jejuni*); *Cc* (*C. coli*); *C.* spp. (Unspecified species of the genus *Campylobacter*).

Here we describe the genetic basis of CIP resistance in members of the genus *Campylobacter* and the mechanisms of emergence and spread among strains and species. Drawing on recent publications that describe CIP-resistance in isolates from a number of different sources and countries, we assess if resistance in members of the genus *Campylobacter* is still rising and review the potential for using DNA sequence-based approaches to predict FQ-resistance in members of the genus *Campylobacter.*

### Mechanisms of resistance

In *Campylobacter*, FQs work by inhibiting a large enzyme, DNA gyrase, that is involved in DNA replication and transcription [5, 14, 26, 27]. It is now commonly recognised that the most frequent mechanism of CIP resistance in members of the genus *Campylobacter* is a single point mutation C257T in the *gyrA* gene, within the QRDR. This results in an amino acid substitution in the Gyrase A subunit at position 86, from threonine to isoleucine [5, 14, 28–31] and has been reported in all CIP-resistant strains of *C. jejuni* isolated from clinical samples in an example study [32]. Other mutations within the *gry*A gene have been associated with increased resistance to ciprofloxacin but at lower concentrations and frequency [5, 14, 32, 33]. The g*yr*A mutation works synergistically with the most common *Campylobacter* drug efflux pump CmeABC, where, when expression is elevated, the emergence of FQ-resistant strains is increased [5, 14]. However, in the absence of the *gyr*A gene mutation, over-expression of the CmeABC efflux pump does not generate ciprofloxacin resistance [5, 28, 32, 34, 35]. Other factors enhance the level of resistance further, such as the 16 bp inverted repeat (IR) in the *cme*R–*cme*ABC intergenic region. When this mutation occurs in conjunction with the C257T-*gyr*A mutation, the proportion of resistant isolates increases and a higher mean ciprofloxacin minimum inhibitory concentration (MIC) is achieved. For example, in culture at a CIP concentration of 64–512 µg ml^−1^, 30 % resistance was observed in C257T-*gyr*A mutants compared with 97 % among IR-C257T mutants [28]. In addition a variant of the *cme*ABC gene that enhances CIP resistance (RE-*cme*ABC) has recently been identified and is increasing in prevalence [34].

Variation in other genes can also indirectly influence the level of CIP resistance. For example, variations in the mutant frequency decline gene (*mfd*) may have a role, since the inactivation of this gene has been shown to reduce mutations 100-fold [5, 36]. Unlike the more prolonged and often stepwise selection process for macrolide resistance [5], CIP resistance can accumulate rapidly in the population through mutation in different strains of members of the genus *Campylobacter* and selection pressure enriching for isolates that have a resistance mutations [14, 18, 27, 37].

### Acquisition and spread of resistance

Spontaneous mutation is a major mechanism for acquisition of FQ-resistance. In an environment where resistance confers a selective advantage, clonal reproduction among resistant lineages will lead to local expansion. Consistent with this, there is evidence that CIP resistance phenotypes are more common among certain lineages or clonal complexes (CC) [38]. This lineage association has been observed among isolates from UK retail poultry [38] and clinical samples [39], and several studies have associated ciprofloxacin resistance with certain lineages of members of the genus *Campylobacter*, including CC-21, CC-206, CC-353 and CC-354 [40–45]. However, in contrasting studies in China there was no association between CC and CIP-resistance phenotypes, indicating that the acquisition of resistance had occurred in numerous distantly related strains of members of the genus *Campylobacter* [28, 46]. This indicates widespread dispersed resistance rather than clonal expansion of resistant strains. One explanation for this is the spread of resistance between strains by horizontal gene transfer (HGT) [5, 18].

In members of the genus *Campylobacter*, FQ-resistance is encoded on the chromosome and may therefore be expected to be less transmissible between lineages than AMR that are encoded on highly mobile plasmids. However, in highly recombining bacteria such as *C. jejuni* and *C. coli,* there is frequent natural transformation [5, 14] that may facilitate the spread of resistance genes. It is difficult to separate the role of mutation and HGT in conferring quinolone resistant phenotypes from DNA sequence data alone, but the distribution of resistance among relatively distant lineages (clonal complexes, CC’s) is consistent with widespread acquisition by mutation and recombination. The most likely evolutionary scenario is that CIP-resistance originates from independent point mutations in the *gyr*A gene or horizontal acquisition of resistance-encoding sequence(s). Mutants may proliferate locally but since the *gyr*A mutation does not incur a strong fitness cost on the recipient genotype, it persists in the absence of selective pressure [14]. When compared with *C. jejuni*, *C. coli* tends to have a greater proportion of isolates that are resistant to CIP, along with other AM’s [4, 6, 7, 15, 16]. There are several potential reasons for the higher FQ resistance in *C. coli.* First, specific mutations or natural transformations may occur at higher frequencies. For example, strains belonging to the *C. coli* 828 clonal complex show evidence of extremely high levels of interspecies recombination, with around 10 % of the genome introgressed from *C. jejuni* [47, 48]. Second, it is also possible that uncharacterized genes or adaptations may be present in *C. coli,* such as those associated with gentamicin resistance [49]. Third, the majority of *C. coli* isolated from clinical and agricultural sources belong to a single clonal complex (the ST-828 complex). Resistance mechanisms associated with this expansion will be shared by a large proportion of isolated strains. A more detailed understanding of the development and maintenance of AMR in strains and species of the genus *Campylobacter* will dependent upon analysis of exposure, transmission, strain mutation/recombination frequency and the fitness cost of adaptations to different AMs in the absence of selective pressure [7].

### Predicting resistance from whole-genome data

Advances in whole-genome sequencing (WGS) technology and analysis have greatly improved understanding of the genetic basis of phenotypic variation. Large numbers of genomes of members of the genus *Campylobacter* are now routinely sequenced, and this has considerable potential for improving understanding of the evolution of FQ resistance [5]. A recent study correlated multiple *in vitro* antimicrobial resistance phenotypes with WGS genotype data [50]. Predictions based upon genomic variation were >99 % accurate, indicating that WGS may be a powerful tool for AMR surveillance programs. Other sequence-based approaches have also accurately detected the single point mutation in the *gyr*A gene in phenotypically confirmed CIP-resistant members of the genus *Campylobacter* [24] and the European Committee for Antimicrobial Susceptibility Testing (EUCAST) is currently reviewing the use and accuracy of WGS as a predictor of AMR [51] for surveillance and monitoring programs.

**Fig. 1.**
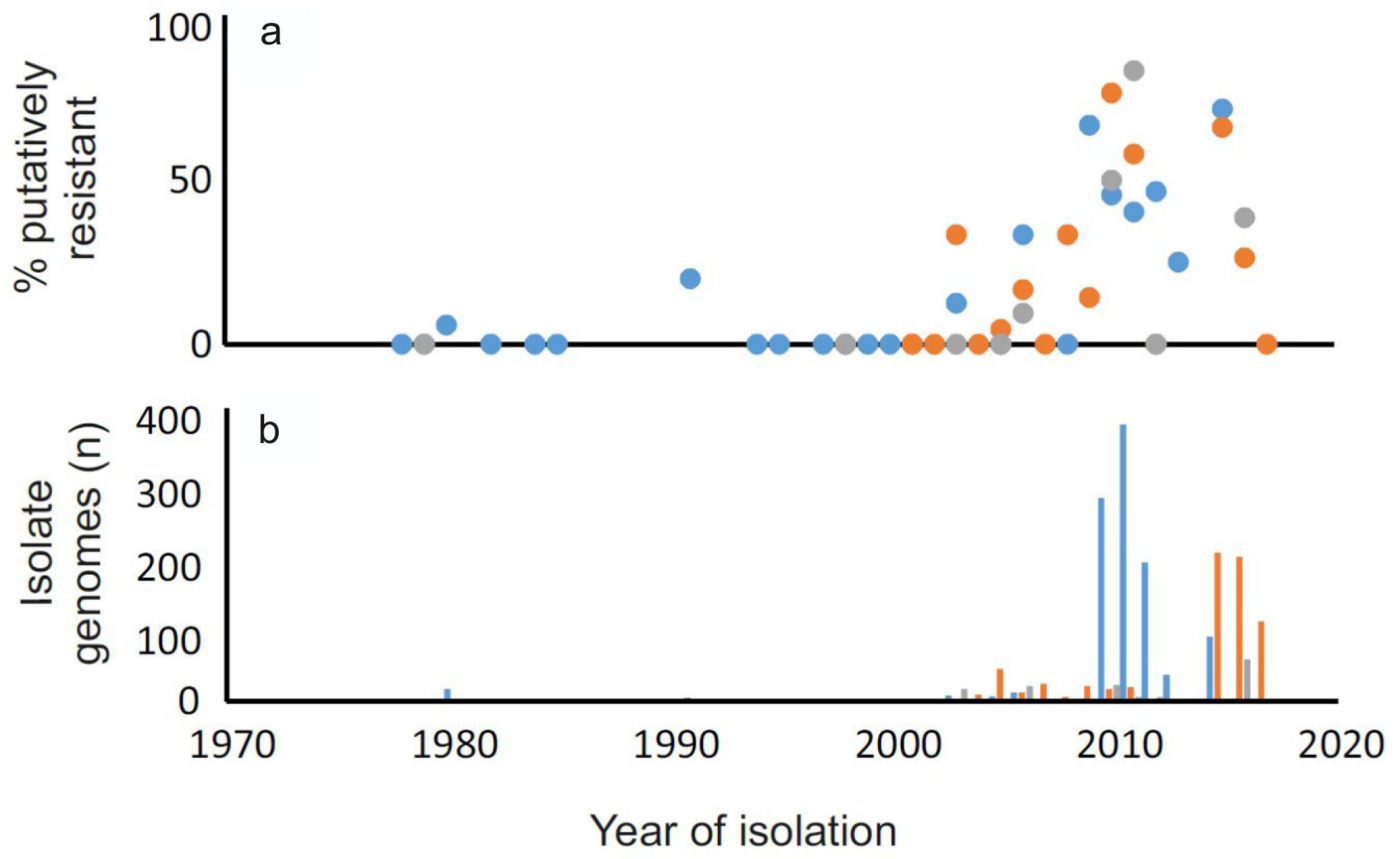
Proportion of genomes of isolates of members of the genus *Campylobacter* containing putative ciprofloxacin resistance. Isolate genomes that contained a complete *gyr*A gene sequence from 1038 human (Blue), 670 chicken (Orange) and 136 ruminant (Grey) samples collected from 1978 to 2017 were compared. (a) The percentage of isolates containing isoleucine at amino acid position 86 was determined and (b) the total number of samples was recorded.

To demonstrate the utility of studying putative CIP resistance using genome data, we analysed assembled draft genomes archived on the Sheppardlab BIGSdb [52] (Table S1, available in the online version of this article). Briefly, *gyr*A allelic variants were identified among isolate genomes from human (clinical), chicken (faeces and meat) and ruminant (cattle and sheep faeces and meat) samples. Gene homology was defined using blast, with those found to have >70 % nucleotide identity in >50 % of the continuous sequence length, considered to be homologous. While the number of homologues identified would vary depending on the identity threshold, we used these as they are the default settings within the database [52]. Putative resistance genes and the proportion containing threonine and isoleucine at amino acid position 86 were compared after alignment using the mega program, version 7.0. This allowed comparison of putative CIP resistance in the genomes of 1844 isolates of members of the genus *Campylobacter* (Fig. 1). This demonstrates the utility of WGS for making predictions about resistance phenotypes on the basis of sequence variation in genes for which the putative function is known. There is some evidence for increasing resistance over time among strains from humans, chickens and ruminants but this is not a structured study so further analysis would be needed.

### Ciprofloxacin resistance among clinical isolates

Estimates of the proportion of CIP-resistant isolates of members of the genus *Campylobacter* from clinical samples vary considerably between published studies [6, 15, 17, 25, 39, 53–57] (Table 1). An increasing trend in the proportion of clinical CIP-resistant isolates of members of the genus *Campylobacter* has been reported in 2015 for 5 out of 17 (for *C. jejuni*) and 2 out of 13 (for *C. coli*) European member states [15]. In England, Scotland and Wales, CIP-resistant members of the genus *Campylobacter* increased from 7 % of clinical isolates in 1995 to 38 % in 2008, with a similar trend observed among *C. jejuni* in Northern Ireland (from 9 % in 1996 to 32 % in 2007) [58, 59]. While direct contextualization with isolates from the USA is not possible because of differences in data collection, the proportion of CIP-resistant clinical isolates was seen to increase from 22 % (2004–2010) to 25 % (2011–2012) [55] with a similar level of resistance observed in *C. jejuni* from human infections reported in Canada (2002–2014) [17].

Reports of reductions in the proportion of CIP-resistant members of the genus *Campylobacter* from human infections are comparatively rare. None of the EU countries with AMR data covering the previous three years (2013–2015) reported declining trends of CIP resistance in isolates of members of the genus *Campylobacter* from humans [15]. Furthermore, the members of the EU and the USA are thought to be among the countries with a lower resistance burden, and global trends indicate a higher proportion of CIP resistance in other countries. For example, among clinical samples from China a sustained increase has led to a rise of CIP resistance in *C. jejuni* from 78 % (1994–2002) to 90 % (2003–2010) [25]. Consistent with this, foreign travel is a major risk factor for infection with FQ-resistant *Campylobacter*. Studies in Canada, the USA and the UK revealed that individuals that had travelled abroad were more likely to have CIP-resistant strains [17, 39, 55]. In the USA 62.4 % of infections with members of the genus *Campylobacter*acquired abroad were CIP-resistant compared with 14.4 % of domestically acquired infections and CIP-resistant strains were five times more likely to have been acquired abroad than domestically in the UK [39, 55].

### Ciprofloxacin resistance in livestock

Consistent with the high levels of FQ resistance in clinical samples, numerous studies have reported CIP-resistant members of the genus *Campylobacter* in livestock, including ruminants [33, 40, 60], swine [15, 46, 60], poultry [6, 24, 46, 61, 62] and poultry retail meat [6, 16, 63, 64] (Table 1). In cattle from the USA, 16 % of *C. jejuni* isolated from gut and faecal samples (2014) were resistant to CIP [40] and in isolates from faeces and carcasses of cattle from Africa (2013–2014), the proportion of CIP-resistant species of the genus *Campylobacter* was 43 % [60]. Resistance was even more common among *C. coli* from fattening pigs in Europe (2015) with 62 % of isolates displaying CIP resistance [15].

As in clinical samples over a similar period, there is also evidence for an increasing trend in CIP-resistant members of the genus *Campylobacter* from beef and dairy cattle in the USA (2013–2014) [4] and broiler chickens from various European countries (2008–2014) [6]. In Austria and Spain the proportion of CIP-resistant *C. jejuni* from broilers remained stable over this period, as did resistance in *C. coli* isolated in France, although they remained at a relatively high prevalence. The only European country in which a reduction in the number of CIP-resistant *C. jejuni* and *C. coli* isolated from broilers was observed over this period (2008–2014) was the Netherlands [6].

### Potential for spread of resistance from animals to humans

The transmission of FQ-resistant bacteria from agricultural animals to humans is difficult to prove and a recent global report on surveillance of antimicrobial resistance emphasised the need to collect more data on the effects of AMR in foodborne bacteria on animal and human health [21, 65]. Currently, there is little direct evidence of the transmission of FQ-resistant bacteria within livestock and to humans via food. The occurrence of resistant members of the genus *Campylobacter* on retail poultry meat is a major concern since this is a principal source of isolates infecting humans [3, 66]. Data covering the last three years reveals an overall increase in CIP-resistance among both C*. jejuni* and *C. coli* isolates from chicken meat [6]. In the USA, CIP-resistance among isolates from chicken breast meat increased from 15 to 17 % (*C. jejuni*) and from 10 to 26 % (*C. coli*) from 2002 to 2007 [67], and a separate study recorded a similar rise among *C. jejuni* isolates between 2013 and 2014 [4]. In UK studies, the proportion of CIP-resistant *C. jejuni* and *C. coli* isolated from retail chicken showed a similar increase between 2007–2008 and 2014–2015 with the proportion of resistant *C. jejuni* and *C. coli* isolates increasing from 21 to 49 % and from 35 to 53 %, respectively [16, 68]. Interestingly, samples from frozen chicken were more likely to contain ciprofloxacin-resistant isolates [68]

It has been noted that the proportion of CIP-resistant members of the genus *Campylobacter* in poultry meat is often strikingly similar to the proportion observed in human clinical cases [6, 16, 58, 69]. In China, broilers and chicken meat showed the highest *Campylobacter* CIP resistance at 99 to 100 %, which corresponds to the extremely high levels recorded in human isolates at 90 % between 2003 and 2010 [25, 61, 64]. Likewise, in Poland 66 % of human clinical isolates of members of the genus *Campylobacter* were CIP-resistant, which was similar to the 67 % sampled from chicken meat (Table 1) [63]. In the case where the direct consumption of FQ-resistant bacteria leads to human infection, it is compelling to conclude that the use of antimicrobials to treat animals potentially erodes the efficacy of important human drug treatments. The antimicrobial resistance in *C. jejuni* and *C. coli* of both animal and human origin, has a strong correlation with the amount of antimicrobial use in the animal production system (mg per kg of meat produced). Those with stricter guidelines tend to have lower proportions of resistant isolates (e.g. Nordic countries) [7, 70]. However, using WGS to identify the *gyr*A mutations in isolates of members of the genus *Campylobacter* from poultry failed to cluster isolates according to the country of origin or the current use of FQ in livestock production [45]. In addition, data from the USA Centre for Disease Control National Antimicrobial Resistance Monitoring System database [54] indicates that the 2005 USA ban on the use of FQ’s in livestock has had little effect on the increasing FQ-resistance among *C. jejuni* isolates from clinical samples.

The fate of genomic variation associated with resistance is at least partially determined by fitness. Substitutions that impose little or no fitness cost on the cell have a higher probability of persisting in the absence of antibiotic treatment [71]. It is known that FQs impose a fitness cost in members of the genus *Campylobacter* but this can vary depending on strain and study conditions, with resistance potentially persisting for some time in the absence of FQs [72], potentially associated with compensatory mutations that alleviate the costs of resistance. This means that livestock may not just act as transient vehicles of FQ resistance but are dissemination points where members of the genus *Campylobacter* resistant to FQ’s can persist and circulate within the animal population for several years following the decreased use of AM’s [7]. However, production systems that use AMs at a higher rate present an increased risk of resistant isolates spreading, at least on a local scale. Monitoring of AMR is seriously lacking in many countries [21] where this, along with different practices and regulations, is associated with wide variation in AMR between different countries [7]. Because of trade networks, global implementation of monitoring and restriction of the use of AM’s in livestock would be beneficial in the fight against AMR, with more detailed information on AM usage at local, regional and national scales to more accurately assess the potential risk of transfer to humans [7, 21, 70].

### Conclusions

The mechanisms by which FQ-resistance is acquired in *Campylobacter* (mutation and HGT) are well established, and routine surveillance of resistance phenotypes in clinical samples describe a continued and sustained increase in many countries. This trend is mirrored in isolates from livestock, especially chickens, which are a major source of human disease. It is difficult to quantify the extent to which the use of antimicrobials in agriculture reduces the efficacy of drugs such as fluoroquinolones in treating human infections. This is exacerbated by the slow rate at which FQ-resistance in *Campylobacter* is purged in the absence of AMs, meaning that, once acquired, resistance can be maintained in populations despite restrictions on the use of FQ’s in animal production. WGS offers a potential tool for improving understanding of the emergence and maintenance of AMR among members of the genus *Campylobacter* from multiple host species. On the basis of AMR phenotype predictions from large population genomic datasets it will be possible to more accurately characterize source and sink populations, environmental reservoirs and specific microevolutionary events associated with acquisition, maintenance and spread of resistance.

## Data bibliography

1. Supporting external data for the prediction of putative CIP resistance using genome data is included as a supplementary table (Table S1) listing the isolates and a supplementary data file of *gyrA* sequences from all isolates.
